# Prognostic Factors and a Predictive Nomogram of Cancer-Specific Survival of Epithelial Ovarian Cancer Patients with Pelvic Exenteration Treatment

**DOI:** 10.1155/2023/9219067

**Published:** 2023-08-17

**Authors:** Ting Wang, Xin Fu, Lei Zhang, Shuna Liu, Ziqi Tao, Fang Wang

**Affiliations:** ^1^Department of Laboratory Medicine, The First Affiliated Hospital of Nanjing Medical University, Nanjing 210029, China; ^2^Branch of National Clinical Research Center for Laboratory Medicine, Nanjing 210029, China; ^3^Jiangsu Provincial Medical Key Discipline, Nanjing 210029, China; ^4^Clinical Laboratory, Baoshan People's Hospital, Baoshan, Yunnan 678000, China; ^5^Department of Gynecology, The Affiliated Huaian No. 1 People's Hospital of Nanjing Medical University, Huaian 223300, China

## Abstract

**Objective:**

The aim of this study was to explore prognostic factors, develop and internally validate a prognostic nomogram model, and predict the cancer-specific survival (CCS) of epithelial ovarian cancer (EOC) patients with pelvic exenteration (PE) treatment.

**Methods:**

A total of 454 EOC patients from the Surveillance, Epidemiology, and End Results (SEER) database were collected according to the inclusion criteria and randomly divided into the training (*n* = 317) and validation (*n* = 137) cohorts. Prognostic factors of EOC patients with PE treatment were explored by univariate and multivariate stepwise Cox regression analyses. A predictive nomogram was constructed based on selected risk factors. The predictive power of the constructed nomogram was assessed by the time-dependent receiver operating characteristic (ROC) curve. Kaplan–Meier (KM) curve stratified by patients' nomoscore was also plotted to assess the risk stratification of the established nomogram. In internal validation, the C index, calibration curve, and decision curve analysis (DCA) were employed to assess the discrimination, calibration, and clinical utility of the models, respectively.

**Results:**

In the training cohort, age, histological type, Federation of Gynecology and Obstetrics (FIGO) stage, number of examined lymph nodes, and number of positive lymph nodes were found to be independent prognostic factors of postoperative CSS. A practical nomogram model of EOC patients with PE treatment was constructed based on these selected risk factors. Time-dependent ROC curves and KM curves showed the superior predictive capability and excellent clinical stratification of the nomogram in both training and validation cohorts. In the internal validation, the C index, calibration plots, and DCA in the training and validation cohorts confirmed that the nomogram presents a high level of prediction accuracy and clinical applicability.

**Conclusion:**

Our nomogram exhibited satisfactory survival prediction and prognostic discrimination. It is a user-friendly tool with high clinical pragmatism for estimating prognosis and guiding the long-term management of EOC patients with PE treatment.

## 1. Introduction

Ovarian cancer (OC) is an aggressive disease characterized by its occult property and high rate of recurrence, making iterative cytoreduction a potentially beneficial approach. Among OC cases, epithelial ovarian cancer (EOC) accounts for approximately 90% of cases and represents the most prevalent histological type [1]. To achieve optimal cytoreduction for OC, pelvic exenteration (PE) has been proposed and implemented [2]. PE is a radical surgical intervention involving the removal of multiple organs in the pelvis, such as the reproductive organs, lower urinary tract, rectum, part of the colon and anus, and lymph nodes in the pelvis [3]. Despite carrying the elevated risk of death, PE may offer a last resort for patients with recurrent or advanced OC that cannot be resected with a lesser extent of operation [4]. Recently, with advances in perioperative care and surgical techniques, the perioperative morbidity of PE has continued to decline. Consequently, PE, as a potential optimizing surgical cytoreduction approach for pelvic malignancies, attracts significant attention in EOC therapy [5].

While previous studies have demonstrated that several clinical parameters, such as age, distant metastases, and histological type, are important prognostic factors for EOC patients, however, their impact on the postoperative survival in patients undergoing PE treatment remains inconclusive [6–8]. Lymph node status is presently recognized as a crucial component of the postoperative risk stratification system for EOC patients [9]. Although being incorporated into the FIGO staging system, positive lymph node may not adequately reflect actual lymph node status in that the number of examined lymph node is not taken into consideration. The effect of actual lymph node status on postoperative outcomes of EOC patients with PE treatment is worthy to be assessed. Furthermore, it is still controversial whether adjuvant chemotherapy is necessary after the pelvic cavity [10–12]. Therefore, the purpose of this study was to explore the risk factors affecting postoperative survival and to establish an accurate prognostic model for EOC patients with PE treatment based on the SEER database, which collects clinical information of approximately 28% U.S. population. We aimed to provide valuable insights that would enable doctors to develop personalized treatment and follow-up strategies for patients with EOC.

Nomogram is a user-friendly visualization tool of models to predict and quantify patient survival [13]. By integrating various prognostic variables, nomograms generate individual numerical probabilities of clinical events and intuitively present complex mathematical formulas in the form of intuitive visual diagrams [14]. Compared with the FIGO staging system, the nomogram satisfies our pursuit of personalized prognosis assessment. However, to the best of our knowledge, there is currently no specific nomogram available for OC patients with PE treatment. Hence, in this study, we aimed to identify potential prognostic factors for postoperative survival in EOC patients with PE treatment, employing univariate and multivariate stepwise Cox regression analyses based on the SEER database. Subsequently, a nomogram model was developed and validated in both the training and validation cohorts using the identified independent risk factors to predict CSS of EOC patients with PE treatment.

## 2. Patients and Method

### 2.1. Study Cohort Selection

Patient information was obtained using SEER *∗* Stat (version 8.4.0.1) from SEER Program Database (Incidence—SEER Research Plus Data, 17 Registries, released April 2022, based on the November 2021 submission). Cases of OC (International Classification of Diseases (ICD)-O-3, primary site, C56.9: ovary) diagnosed between 2004 and 2015 were obtained. To select epithelial histologic type, the ICD-O-3 morphology codes “8020–8022, 8441–8442, 8460–8463, 9014”; “8470–8472, 8480-8481, 9015”; “8380–8383, 8570”; and “8290, 8310, 8313, 8443–8444” were used to identify women with serous, mucinous, endometrioid, and clear cell ovarian tumors, respectively. The inclusion criteria of research objects were as follows: only one primary malignant tumor; undergoing PE treatment; survival ≥1 month; histology-confirmed diagnosis; with known and complete lymph node status and TNM stage. Patients with the following characteristics were excluded: diagnosed only through autopsy and death certificate; with nodes aspiration; unknown liver or lung metastasis status (for patients from 2010 to 2015). Finally, a total of 454 eligible patients were selected for analysis in this study. The outcome in this study was CSS, which was defined as the time interval between the time of diagnosis and EOC-caused death. In addition, the FIGO staging system of patients was redefined based on the 6th (2004–2009) and 7th (2010–2015) editions of AJCC TNM staging according to the NCCN Manual (2015). All cases were randomly divided into training cohort (*n* = 317) and validation cohort (*n* = 137) with a ratio of 3 : 1 for corresponding variables selection, as well as nomogram construction and validation [6, 15].

### 2.2. Variable Collection and Reclassification

The diagnosis and treatment process of EOC patients in the SEER database in the past period were reviewed to identify prognostic factors for CSS. We obtained the following variables of selected patients from the SEER database directly: age at diagnosis, race, year of diagnosis, marital status, histological type, tumor grade, tumor laterality, FIGO stage, clinical AJCC *T* stage, N stage, *M* stage, surgical approach, radiotherapy, chemotherapy, residual tumor volume, liver metastasis, lung metastasis, CA125, lymph nodes examined, lymph nodes positive, and tumor size. In addition, LNR (the ratio between lymph nodes examined and lymph nodes positive) and LODDS (log of odds between the number of positive lymph nodes and negative lymph nodes) were indirectly obtained by calculation. Variables including residual tumor volume, liver metastasis, and lung metastasis started to be collected from 2010; therefore, these variables were only obtained for 220 cases (2010–2015). In the SEER database, the age, lymph nodes examined, lymph nodes positive, and tumor size were recorded as continuous variables. The others were recorded as categorical variables. Several variables were reclassified. Patients in American Indian/Alaskan Native and Asian/Pacific Islanders were recorded as “other” under race; patients in widowed, divorced, separated, unmarried, or domestic partners were recorded as “other” under marital status. Grade 1 indicated well differentiated when Grade 2 indicated mean medium differentiated, and Grade 3 indicated poorly differentiated and undifferentiated. The way of displaying data for categorical variables was count and percentage.

### 2.3. Statistical Analysis

All statistical analysis was performed using *R* Version 4.2.1 (R Foundation, Vienna, Austria, https://www.r-project.org) in the *R* Studio environment. Variance inflation factor (VIF) was calculated and plotted using “performance” and “see” *R* package. Continuous variables were transformed into categorical variables by optimal cutoff values which were determined using the X-tile software (https://tissuearray.org/) [16]. Univariate Cox regression analysis and multivariate Cox proportional hazard regression analysis (forward stepwise selection methods) were applied to evaluate the association between different potential prognostic variables and CSS using “survival” and “finalfit” *R* packages. Nomograms of 3-year and 5-year CSS were developed based on the selected independent prognostic factors identified from the multivariate analysis using “regplot” *R* package. The predictive performance of the nomogram was measured by the area under the time-dependent ROC curve, and risk stratification was presented by Kaplan–Meier curve using “survivalROC” and “survminer” *R* packages, respectively. Bootstrap with a resample of 1000 times was performed in corresponding internal validation. The discrimination of the constructed nomogram was measured by the C index using “riskRegression” package. Then, the calibration curve which could reflect the extent to which a model correctly estimates the absolute risk was plotted using “rms” *R* package. Improvement of patient outcome, which benefits from nomogram-assisted decisions, was judged by being compared to default strategies of treating all or no patients in the decision curve analysis (DCA) using “ggDCA” package. All tests were two-sided, and *P* < 0.05 was considered statistically significant.

## 3. Results

### 3.1. Demographic and Clinical Characteristics

**Figure 1:**
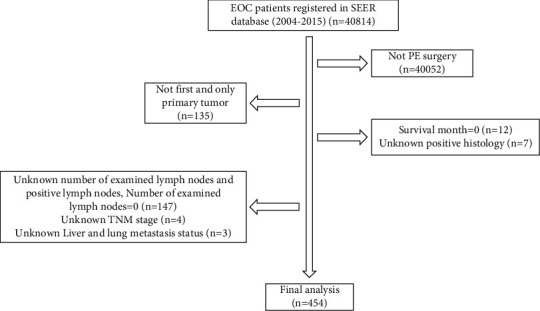
Patient selection flowchart.

The detailed process of patient selection is shown in Figure 1. All patients were randomly divided into training cohort and validation cohort with a ratio of 3 : 1 utilizing *R* software. The results showed that there were no differences between training cohort and validation cohort (Table 1, *P* > 0.05). The demographic and clinical characteristics of all patients are listed in Table 1. Among all cases identified in the database, the majority (83.3%) of the patients were aged younger than 70 years old. More than half (60.6%) of the patients report tumor originated from bilateral ovary, grade III (73.8%), and serous histologic type (89.4%). Most subjects were in stages III and IV (93.8%). Posterior pelvic exenteration (PPE) accounts for 78.6% of treatments for patients. Most of the patients had chemotherapy (82.4%) as the standard treatment, and only 1.1% of patients had radiotherapy. In terms of diagnosis at lymph nodes, tumor size, and CA125, less than 9 positive lymph nodes (78.4%), larger than 38 mm (63.4%), and CA125 positive (81.5%) had the highest percentage.

### 3.2. Optimal Cutoff of Age, Lymph Nodes, and Tumor Size

**Figure 2:**
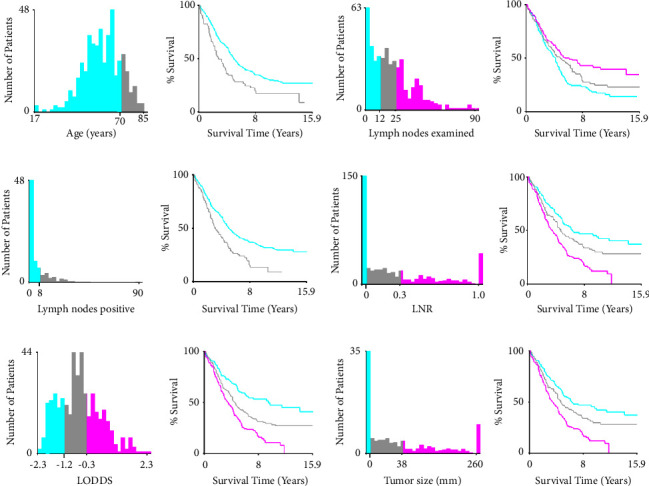
Optimal cutoff values determination of continuous variables and Kaplan–Meier curves stratified by the values using x-tile software. (a) Ages were split into two groups: ≤70 years old and ≥71 years old; (b) cutoff values of lymph nodes examined were 12 nodes and 25 nodes; (c) cutoff value of lymph nodes positive was 8 nodes; (d) LNR were categorized into three subgroups: (0, 0.03), (0.03, 0.32), and (0.32, 1.00). (e) LODDS were divided into three groups: (−2.25, −1.18), (−1.18, −0.25), and (−0.25, 2.26). (f) Threshold of tumor size was 38 mm.

Multicollinearity bias between the number of positive lymph nodes and lymph nodes examined was first evaluated by calculating the variance inflation factor (VIF). As is shown in Supplementary Figure 1, the VIF values for both the number of lymph nodes examined and the number of positive lymph nodes were less than 2, indicating the absence of collinearity between the two variables. To further investigate the role of continuous variables (age, lymph nodes, and tumor size) on prognosis, we used X-tile to present a histogram of the data distribution and Kaplan–Meier curves in which data were stratified by the optimal cutoff values. For age, the best threshold was 70 years old, and the older the people, the worse their survival (Figure 2(a)). Meanwhile, lymph nodes examined were categorized into three subgroups: 1∼12, 13∼25, and ≥26 (Figure 2(b)). It could be found that more regional lymph nodes examined indicated a favorable survival. Lymph nodes positive were split into two groups (0∼8 and ≥9), and more positive lymph nodes indicated worse survival (Figure 2(c)). Similarly, the best cutoff values of LNR were 0.03 and 0.32, and larger LNR indicated worse survival (Figure 2(d)). In addition, the threshold was 1.18 and −0.25 for LODDS, and larger LODDS indicated worse survival (Figure 2(e)). The optional threshold was 38 mm for tumor size, and the patients with tumor smaller than 38 mm had a better survival than that larger than 38 mm (Figure 2(f)).

### 3.3. Independent Predictors Analysis for CSS of EOC Patients with PE Treatment

The univariate and multivariate stepwise Cox regression models were performed in the training cohort (*n* = 317) first to identify the prognostic factors of predicting CSS of EOC patients with PE treatment. Results showed that age, histology type, FIGO stage, lymph nodes examined, and lymph nodes positive were independent prognostic factors (*P* < 0.05) (Table 2). The results of multivariate stepwise Cox regression analysis were also displayed by forest plot (Supplementary Figure 2). Then, prognosis factors were further explored in 2010–2015 period cases (*n* = 220) which subset from overall cases (2004–2015, *n* = 454). In addition to histology type, FIGO stage, lymph nodes examined, and lymph nodes positive which had been identified in the above analysis, the univariate Cox analysis revealed that residual tumor volume was a potential risk factor for CSS (*P* < 0.05). However, residual tumor volume was found not associated with CSS (*P* > 0.05) by multivariate Cox analysis. The detailed univariate and multivariate analysis results of selected variables are presented in Supplementary Table 1.

### 3.4. Development of a Nomogram of CSS in Training Cohort

**Figure 3:**
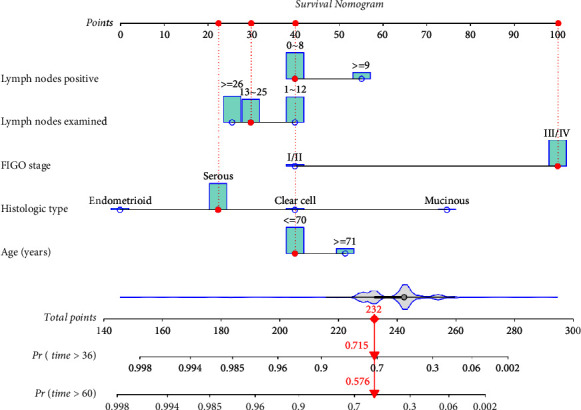
Nomogram to predict 3- and 5-year CSS for EOC patients with PE treatment. The points of each patient could be calculated by adding the assigned points of each characteristic. For example, the red points and dotted line on this graph show the corresponding scores of selected characteristics of each variable. The indicated survival probability of this patient is 71.5% for 3 years and 57.6% for 5 years based on the total point of 232.

The nomogram was constructed by incorporating the above-identified independent prognostic variables to predict the 3-, and 5-year CSS in EOC patients with PE treatment (Figure 3). The estimated probability of CSS at 3 and 5 years could be determined by summing the score of each variable which was assigned a score ranging from 0 to 100. The nomograms demonstrated that the FIGO stage and histologic type contributed the most to CSS for EOC patients with PE treatment. Besides, the exact score concerning each prognosis factor is presented in Supplementary Table 2. In addition, the number of examined lymph nodes was found to have an inverse relationship with its corresponding score.

### 3.5. Validation of Nomogram in Training Cohort and Validation Cohort

**Figure 4:**
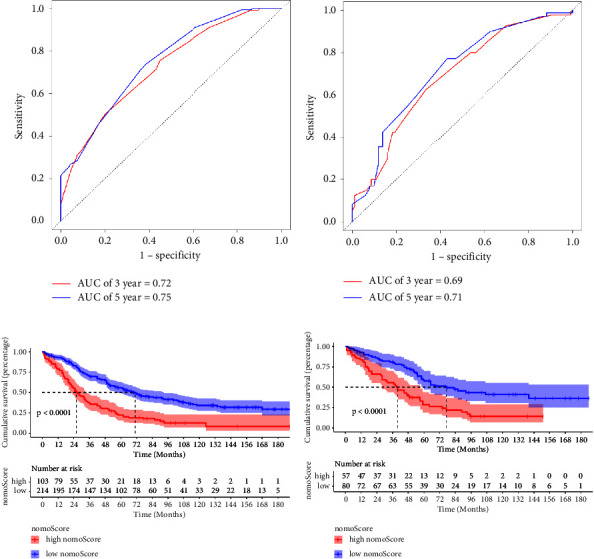
ROC curves and Kaplan–Meier curves of the nomogram. ROC curves of the constructed nomogram in the training cohort (a) and validation cohort (b) at 3 years (blue line) and 5 years (red line). (c, d) The patients were divided into two groups: ≤ 242 points (low nomoscore group) and >242 points (high nomoscore group). The Kaplan–Meier survival curves of the patients in the training cohort (c) and in the validation cohort (d) were plotted. *P* value of log-rank test showed the difference of significance among survival of two subgroups. Dotted lines indicate the median survival time of subgroups.

The discrimination of the nomogram was assessed by time-dependent ROC analysis in both the training cohort and validation cohort (Figures 4(a) and 4(b)). ROC analysis showed that the AUCs of the nomogram in the training cohort for the 3 and 5 years reached 0.72 and 0.75, while 3- and 5-year AUCs of the nomogram in the validation cohort are 0.69 and 0.71. In addition, AUCs of nomogram varying both the training cohort and validation cohort with time are shown in Supplementary Figures 3A and 3B. Then, the ability of the risk stratification was evaluated in the training cohort and validation cohort. The patients were split into two subgroups based on the median risk score (nomoscore) which was calculated from the nomogram for further assessment of the clinical utility. In both training cohort and validation cohort, the median of nomoscore is 242, and Kaplan–Meier curves of the probability of CSS showed that log-rank *P* values between survival curves of two subgroups were statistically significant (*P* < 0.0001) (Figures 4(c) and 4(d)). Meanwhile, the median CSS time was significantly longer in the low nomoscore group than in the high nomoscore in both training cohort (71 mo vs. 26 mo) and validation cohort (77 mo vs. 40 mo).

**Figure 5:**
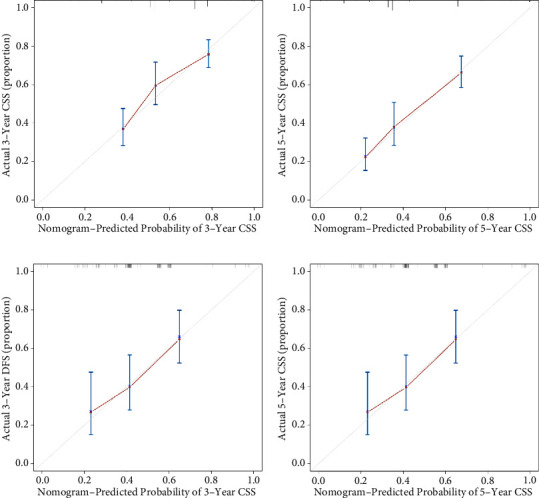
Calibration plot of constructed nomogram for the prediction of 3-year and 5-year CSS. The bootstrapped calibration plot compared predicted probability (*X*-axis) and observed risk (*Y*-axis) at different levels in different groups (3 groups, *B* = 1000) for the prediction of CSS in training cohort (a, b) and validation cohort (c, d). Grey line is the predictional line of an ideal calibration model which is along the 45° diagonal line.

**Figure 6:**
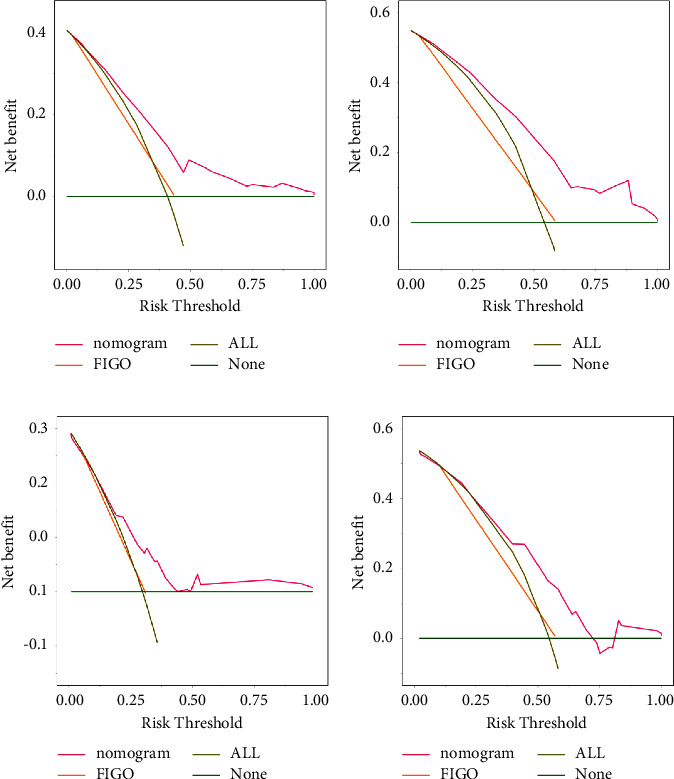
DCA curves of constructed nomogram for the prediction of 3-year and 5-year CSS. The pink line and orange line in DCA curves show the clinical benefit when the clinical strategy was decided by the prediction of the constructed nomogram or FIGO stage in 3-year and 5-year CSS of training cohort (a, b) and validation cohort (c, d), respectively. *X*-axis is threshold probability and *Y*-axis shows the clinical decision net benefits after the benefits minus the disadvantages. “All” indicates the benefit when all patients are treated with clinical interference and “None” indicates the benefit when no patients are treated with clinical interference.

The discrimination of this nomogram was further assessed by the bootstrap validation with 1000 resamplings. The bootstrap-corrected Cindex of the nomogram was 0.72 (95% CI: (0.66, 0.78); 3-year-CSS) and 0.75 (95% CI: (0.70, 0.81); 5-year-CSS) in training cohort and 0.69 (95% CI: (0.60, 0.78); 3-year-CSS) and 0.71 (95% CI: (0.62, 0.80); 5-year-CSS) in validation cohort. The bootstrapped calibration plots for the prediction of 3-year and 5-year CSS in training cohort and validation cohort are shown in Figure 5. The calibration plot of both training cohort and validation cohort showed that the curve was closely fitted with the diagonal line, indicating favorable prediction of the constructed nomogram. Additionally, DCA curves that calculate clinical “net benefit” were plotted to assess the clinic utility of the nomograms and FIGO staging system in prognosis evaluation. The result confirmed that the proposed nomogram would be a superior intervention tool in both training and validation cohorts compared to the strategies of intervening all patients or intervening no one (Figure 6).

## 4. Discussion

Clinical treatment of gynecological recurrent cancer and advanced cancer is very limited. Recently, with the deepening of collaboration of multidisciplinary technology, PE combined with organ function reconstruction has been gradually applied clinically by some doctors in gynecologic malignancies [17]. Many studies had confirmed the efficacy and safety of PE as a feasible surgical option for the treatment of advanced-stage EOC [2, 10–13]. PE, which aims at removing the tumor completely, is proposed as a promising treatment option for EOC to maximize surgical efforts. Considering the increasing popularity of PE in treatment of EOC patients, it is necessary to explore the independent risk factors of postoperative outcomes to guide the long-term management of the EOC patients with PE treatment (such as postoperative follow-up and chemotherapy). Particularly, a model for individual-patient outcome prediction is indispensable and can hopefully maximize the benefit which the patient can gain from PE. In this study, the real information of EOC patients with PE treatment obtained from the SEER database was used to investigate risk factors of survival and establish a prognosis model. Age, histology type, FIGO stage, number of examined lymph nodes, and positive lymph nodes were demonstrated to be independent prognostic factors. Moreover, the constructed nomogram based on these selected variables exhibits excellent performance in discrimination, accuracy, and clinical applicability.

It could be summarized that the majority of overall cases in this study are advanced-stage serous OC patients with treatment of PPE and chemotherapy. It is consistent with some reports that PPE was the most common type of PE and chemotherapy was usually recommended to patients after PE treatment regardless of its uncertain effects [18, 19]. In line with our expectations, clinical pathological characteristics, including age, histologic types, and FIGO stage, which have been consistently demonstrated to be closely correlated with survival of EOC patients with regular debulking surgery or neoadjuvant chemotherapy, were found to be an independent risk factor of CSS of EOC patients with PE treatment [20, 21]. Few studies have revealed the effect of histological subtypes on the prognosis of EOC patients with PE treatment. Our results found that patients of endometrioid, serous, and clear cell subtypes showed better CSS than those of the mucinous subtype. The prognosis of different histological subtypes in EOC has also been controversial and reported to be associated with the FIGO stage. Babaier and Ghatage reported that early-stage mucinous OC presented an excellent prognosis, while advanced-stage mucinous OC disease has a poor outcome which is consistent with findings of Michiel Simons et al. that patients with advanced-stage mucinous OC have a worse prognosis than advanced-stage serous OC (11% vs 26%, *P* < 0.01) [22, 23]. However, Yang et al. demonstrated that patients of serous subtype showed worse CSS than those of the endometrioid, mucinous, and clear cell subtypes [24].

Regarding lymph status, LNR and LODDS have been recently proposed as alternative index for assessing lymph node status in ovarian cancer [7, 25]. Some studies reveal that LNR and LODDS were superior to the number of examined lymph nodes and number of positive lymph nodes in predicting the survival of EOC patients. Intriguingly, in our study, LNR and LODDS were excluded in the process of variable selection which was performed by stepwise Cox regression. Instead of LNR and LODDS, it can be inferred from our results that both the number of lymph nodes examined and lymph nodes positive which make more sense in predicting postoperative outcomes should be simultaneously taken into consideration when formulating an adjuvant treatment plan. In detail, patients with more lymphatic metastasis had worse survival outcomes, and an increased number of lymph nodes examined was associated with improved survival in our study. It could be explained that with the increased number of lymph nodes examined, the probability of retrieving positive lymph nodes will increase, which is critical for staging and determining the need for adjuvant chemotherapy for patients. Large studies have proposed that metastatic disease and CGR (a complete gross resection) were independent prognostic factors after primary cytoreductive surgery for improved progressive-free survival and OS in advanced gynecologic malignancies [2, 11, 26, 27]. Unexpectedly, in this study, univariate Cox regression analysis of 220 cases (2010 to 2015) showed that metastasis of liver and lung which are the most common metastatic sites of EOC was not associated with CSS. It could be explained by the reason that the information in the SEER database was collected at the time of initial diagnosis, which means that the metastasis found latter cannot be recorded. Meanwhile, the results of multivariate Cox analysis showed that the size of residual tumor volume was not associated with CSS, which may be attributed to limited samples. Only 29 clinical cases of R2 (gross residual > 1 cm) were included in the analysis after patient selection, which could interfere with the accuracy and authenticity of the analysis.

Subsequently, a prognostic nomogram model was established for EOC patients with PE treatment based on the selected clinical and pathological factors. Nomograms allow wide application in clinical practice by providing a simplified representation of a complicated statistical model utilizing a user-friendly graphical interface. In this constructed nomogram, patients being more than 70 years old, mucinous histologic type, FIGO stage III/IV, ≥9 examined lymph nodes, and 1-12 positive lymph nodes contributed to high scores, which indicate the low survival probability. In accordance with HR results, the FIGO stage and histological type showed the greatest discriminating power when compared with other variables. Based on the results of the ROC curve and Kaplan–Meier curve, it can be concluded that the nomogram exhibited excellent performance of discrimination in both the training and validation cohorts. As such, this new nomogram model can be used to identify high-risk EOC patients after PE treatment. Furthermore, in internal validation, when the nomogram was applied to the training and validation cohorts, the Cindex and calibration curve indicated decent clinical predictive accuracy, reliability, and repeatability. The results of DCA in our study proved that, compared with the FIGO staging, a constructed nomogram was a well-performing model in clinical applicability. Our nomogram is the first nomogram that is specifically applied to OC patients with PE treatment. Meanwhile, compared to reported studies of OC patients, all variables included in our nomograms are objective and not affected by the subjective evaluation of doctors and the individual feelings of patients [28–30]. Also, all variables could be obtained easily in clinical practice, which facilitated its wide application in clinical practice.

### 4.1. Limitations

First, the SEER database only provides a collection of patients in the United States, and the nomogram constructed in this study has not been demonstrated to be applicable to other regions. Meanwhile, this nomogram was only validated by dividing the total cases into training group and validation group. Therefore, further external validation of the nomogram in totally different independent clinical cases, especially from different countries, is needed to obtain an unbiased estimation. Second, certain factors that might associate with survival were not incorporated into the study because of insufficient information in the SEER database. For example, many potential tumor biomarkers, including HE4 protein and serum mesothelin, were not included in the analysis. The detailed chemotherapy regimens, information on targeted drugs, complications, and other information with important significance for the prognosis of EOC were also not available in the SEER database. Third, our nomogram was established on retrospective data which has an inevitable inherent bias. Also, this study may have selection bias because only patients with specific clinical information were included. Finally, it should be noted that this study had a relatively small sample size of only 454 patients. This limitation in sample size may account for the moderate predictive power observed in our model.

## 5. Conclusion

In summary, we found that age, histological type, FIGO stage, number of examined lymph nodes, and number of positive lymph nodes were independent prognostic factors of CSS in EOC patients with PE treatment. We successfully developed a predictive nomogram of 3-year and 5-year CSS which has been demonstrated to present less bias, superior accuracy, and great clinical value. This high-quality nomogram could provide an important reference for individualized therapeutic suggestions and follow-up strategies.

## Figures and Tables

**Table 1:** Demographic and clinical characteristics of EOC patients included in this study.

	Overall (*N* = 454)	Training group (*N* = 317)	Validation group (*N* = 137)	*P* value
Age (years)				
≤70	378 (83.3%)	268 (84.5%)	110 (80.3%)	0.329
≥71	76 (16.7%)	49 (15.5%)	27 (19.7%)	
Race				
White	393 (86.6%)	276 (87.1%)	117 (85.4%)	0.86
Black	14 (3.1%)	9 (2.8%)	5 (3.6%)	
Others	47 (10.4%)	32 (10.1%)	15 (10.9%)	
Marital status				
Married	272 (59.9%)	186 (58.7%)	86 (62.8%)	0.162
Single	81 (17.8%)	53 (16.7%)	28 (20.4%)	
Others	101 (22.2%)	78 (24.6%)	23 (16.8%)	
Histologic type				
Clear cell	20 (4.4%)	13 (4.1%)	7 (5.1%)	0.819
Endometrioid	21 (4.6%)	14 (4.4%)	7 (5.1%)	
Mucinous	7 (1.5%)	4 (1.3%)	3 (2.2%)	
Serous	406 (89.4%)	286 (90.2%)	120 (87.6%)	
Grade				
I	10 (2.2%)	9 (2.8%)	1 (0.7%)	0.658
II	71 (15.6%)	51 (16.1%)	20 (14.6%)	
III	335(73.8%)	232 (73.2%)	103 (75.2%)	
Unknown	38 (8.4%)	25 (7.9%)	13 (9.5%)	
Laterality				
Bilateral	275 (60.6%)	191 (60.3%)	84 (61.3%)	0.554
Unilateral	156 (34.4%)	112 (35.3%)	44 (32.1%)	
Unknown	23 (5.1%)	14 (4.4%)	9 (6.6%)	
FIGO stage				
I/II	28 (6.2%)	20 (6.3%)	8 (5.8%)	1
III/IV	426 (93.8%)	297 (93.7%)	129 (94.2%)	
T stage				
T1	12 (2.6%)	10 (3.2%)	2 (1.5%)	0.785
T2	26 (5.7%)	18 (5.7%)	8 (5.8%)	
T3	118 (26.0%)	82 (25.9%)	36 (26.3%)	
T4	298 (65.6%)	207 (65.3%)	91 (66.4%)	
N stage				
N0	137 (30.2%)	98 (30.9%)	39 (28.5%)	0.682
N1	317 (69.8%)	219 (69.1%)	98 (71.5%)	
M stage				
M0	272 (59.9%)	194 (61.2%)	78 (56.9%)	0.455
M1	182 (40.1%)	123 (38.8%)	59 (43.1%)	
Surgical approach				
PE	40 (8.8%)	34 (10.7%)	6 (4.4%)	0.188
APE	13 (2.9%)	7 (2.2%)	6 (4.4%)	
PPE	357 (78.6%)	246 (77.6%)	111 (81.0%)	
TPE	38 (8.4%)	26 (8.2%)	12 (8.8%)	
EPE	6 (1.3%)	4 (1.3%)	2 (1.5%)	
Radiotherapy				
No/unknown	449 (98.9%)	314 (99.1%)	135 (98.5%)	1
Yes	5 (1.1%)	3 (0.9%)	2 (1.5%)	
Chemotherapy				
No/unknown	80 (17.6%)	58 (18.3%)	22 (16.1%)	0.66
Yes	374 (82.4%)	259 (81.7%)	115 (83.9%)	
CA125				
Positive	370 (81.5%)	260 (82.0%)	110 (80.3%)	0.484
Negative	16 (3.5%)	9 (2.8%)	7 (5.1%)	
Unknown	68 (15.0%)	48 (15.1%)	20 (14.6%)	
Lymph nodes examined				
1∼12	154 (33.9%)	108 (34.1%)	46 (33.6%)	0.953
13∼25	139 (30.6%)	98 (30.9%)	41 (29.9%)	
≥26	161 (35.5%)	111 (35.0%)	50 (36.5%)	
Lymph nodes positive				
0∼8	356 (78.4%)	254 (80.1%)	102 (74.5%)	0.221
≥9	98 (21.6%)	63 (19.9%)	35 (25.5%)	
LNR				
(0, ≤0.03)	150 (33.0%)	109 (34.4%)	41 (29.9%)	0.651
(>0.03, ≤0.32)	146 (32.2%)	100 (31.5%)	46 (33.6%)	
(>0.32, 1.00)	158 (34.8%)	108 (34.1%)	50 (36.5%)	
LODDS				
(−2.25, ≤−1.18)	124 (27.3%)	91 (28.7%)	33 (24.1%)	0.551
(>−1.18, ≤−0.25)	183 (40.3%)	127 (40.1%)	56 (40.9%)	
(>−0.25, 2.26)	147 (32.4%)	99 (31.2%)	48 (35.0%)	
Tumor size (mm)				
<38	62 (13.7%)	46 (14.5%)	16 (11.7%)	0.245
≥38	288 (63.4%)	205 (64.7%)	83 (60.6%)	
Unknown	104 (22.9%)	66 (20.8%)	38 (27.7%)	

**Table 2:** Univariate and stepwise multivariable Cox regression analysis results of CSS in the training cohort (*n* = 317).

Variables	Univariate analysis			Multivariate analysis		
	HR	95% CI	*P* value	HR	95% CI	*P* value
Age						
≤70 years	1			1		
≥71 years	1.80	1.26–2.57	0.001	2.09	1.45–3.00	<0.001
Histologic type						
Clear cell	1			1		
Endometrioid	0.10	0.02–0.46	<0.001	0.08	0.02–0.37	0.001
Mucinous	1.48	0.40–5.48	0.555	9.27	2.32–37.05	0.002
Serous	0.83	0.42–1.63	0.038	0.32	0.16–0.66	0.002
FIGO stage						
I/II	1			1		
III/IV	25.42	3.56–181.53	0.001	46.88	5.83–376.71	<0.001
Lymph nodes examined						
1∼12	1			1		
13∼25	0.71	0.51–0.98	0.040	0.52	0.37–0.74	<0.001
≥26	0.63	0.46–0.88	0.006	0.40	0.27–0.59	<0.001
Lymph nodes positive						
0∼8	1			1		
≥9	1.85	1.35–2.54	<0.001	2.65	1.8–3.9	<0.001

## Data Availability

The data supporting the findings of this study are available upon request from the corresponding author.

## Supplementary Materials

Supplementary Materials Supplementary Table 1: Univariate and multivariable Cox regression analyses of CSS (*n* = 220). Supplementary Table 2: The points of each characteristic in constructed CSS nomogram. Supplementary Figure 1: VIF values for the number of lymph nodes examined and the number of lymph nodes positive. Supplementary Figure 2: Forest plot of stepwise multivariable Cox regression analysis of CSS in training cohort. Supplementary Figure 3: AUCs varying with the time of the nomogram in the training and validation cohorts. The time-dependent AUCs of constructed nomogram in the training cohort (A) and validation cohort (B) varying from 30 months to 60 months were plotted.
