# Supplementary material for: Prognostic Factors and a Predictive Nomogram of Cancer-Specific Survival of Epithelial Ovarian Cancer Patients with Pelvic Exenteration Treatment
Source: Int J Clin Pract. 2023 Aug 17;2023:9219067. doi: 10.1155/2023/9219067 (PMC10449593; doi:10.1155/2023/9219067)
Supplement: Supplementary Materials — Supplementary Table 1: Univariate and multivariable Cox regression analyses of CSS (n = 220). Supplementary Table 2: The points of each characteristic in constructed CSS nomogram. Supplementary Figure 1: VIF values for the number of lymph nodes examined and the number of lymph nodes positive. Supplementary Figure 2: Forest plot of stepwise multivariable Cox regression analysis of CSS in training cohort. Supplementary Figure 3: AUCs varying with the time of the nomogram in the training and validation cohorts. The time-dependent AUCs of constructed nomogram in the training cohort (A) and validation cohort (B) varying from 30 months to 60 months were plotted. [file 9219067.f1.zip › Supplementary material 1.docx]

**Supplementary Table 1: Univariate and multivariable cox regression analysis of CSS (n=220).**

|  |  | **Univariate analysis** | | |  | **Multivariate analysis** | | |
| --- | --- | --- | --- | --- | --- | --- | --- | --- |
| **Variables** | **N (%)** | **HR** | **95% CI** | **p-value** |  | **HR** | **95% CI** | **p-value** |
| **Age (years)** |  |  |  |  |  |  |  |  |
| <=70 | 182 (82.7) | 1 |  |  |  | 1 |  |  |
| >=71 | 38 (17.3) | 1.47 | 0.95-2.26 | 0.084 |  | 1.40 | 0.89-2.20 | 0.141 |
| **Histologic Type** | |  |  |  |  |  |  |  |
| Clear cell | 8 (3.6) | 1 |  |  |  | 1 |  |  |
| Endometrioid | 10 (4.5) | 0.08 | 0.02-0.41 | 0.002 |  | 0.08 | 0.02-0.39 | 0.002 |
| Mucinous | 2 (0.9) | 17.15 | 3.06-96.08 | 0.001 |  | 18.14 | 2.89-113.91 | 0.002 |
| Serous | 200 (90.9) | 0.27 | 0.12-0.58 | 0.001 |  | 0.14 | 0.06-0.32 | <0.001 |
| **FIGO stage** |  |  |  |  |  |  |  |  |
| I/II | 15 (6.8) | 1 |  |  |  | 1 |  |  |
| III/IV | 205 (93.2) | 5.70 | 1.41-23.06 | 0.015 |  | 5.96 | 1.39-25.56 | 0.016 |
| **Residual tumor volume** | | |  |  |  |  |  |  |
| ≤1cm | 142 (64.5) | 1 |  |  |  | 1 |  |  |
| ＞1cm | 29 (13.2) | 1.79 | 1.13-2.83 | 0.014 |  | 1.37 | 0.84-2.23 | 0.205 |
| unknown | 49 (22.3) | 0.85 | 0.54-1.34 | 0.490 |  | 0.80 | 0.50-1.27 | 0.345 |
| **Liver metastasis** | |  |  |  |  |  |  |  |
| No | 198 (90.0) | 1 |  |  |  |  |  |  |
| Yes | 22 (10.0) | 0.89 | 0.49-1.62 | 0.71 |  |  |  |  |
| **Lung metastasis** | |  |  |  |  |  |  |  |
| No | 207 (94.1) | 1 |  |  |  |  |  |  |
| Yes | 13 (5.9) | 1.23 | 0.62-2.43 | 0.551 |  |  |  |  |
| **Lymph nodes examined** | | |  |  |  |  |  |  |
| 1~12 | 74 (33.6) | 1 |  |  |  | 1 |  |  |
| 13~25 | 63 (28.6) | 0.47 | 0.31-0.74 | 0.001 |  | 0.43 | 0.27-0.69 | <0.001 |
| >=26 | 83 (37.7) | 0.39 | 0.26-0.60 | <0.001 |  | 0.31 | 0.19-0.50 | <0.001 |
| **Lymph nodes positive** | |  |  |  |  |  |  |  |
| 0~8 | 175 (79.5) | 1 |  |  |  | 1 |  |  |
| >=9 | 45 (20.5) | 1.35 | 0.89-2.03 | 0.156 |  | 1.82 | 1.13-2.93 | 0.014 |

**Supplementary Table 2: The points of each characteristic in constructed CSS nomogram.**

| **Characteristic** | **Point** |
| --- | --- |
| **Age (years)** |  |
| <=70 | 40 |
| >=71 | 51 |
| **Histologic type** |  |
| Clear cell | 40 |
| Endometrioid | 0 |
| Mucinous | 75 |
| Serous | 22 |
| **FIGO stage** |  |
| I/II | 40 |
| III/IV | 100 |
| **Lymph nodes examined** | |
| 1~12 | 40 |
| 13~25 | 30 |
| >=26 | 26 |
| **Lymph nodes positive** | |
| 0~8 | 40 |
