# Supplementary material for: Prognostic Factors and a Predictive Nomogram of Cancer-Specific Survival of Epithelial Ovarian Cancer Patients with Pelvic Exenteration Treatment
Source: Int J Clin Pract. 2023 Aug 17;2023:9219067. doi: 10.1155/2023/9219067 (PMC10449593; doi:10.1155/2023/9219067)
Supplement: Supplementary Materials — Supplementary Table 1: Univariate and multivariable Cox regression analyses of CSS (n = 220). Supplementary Table 2: The points of each characteristic in constructed CSS nomogram. Supplementary Figure 1: VIF values for the number of lymph nodes examined and the number of lymph nodes positive. Supplementary Figure 2: Forest plot of stepwise multivariable Cox regression analysis of CSS in training cohort. Supplementary Figure 3: AUCs varying with the time of the nomogram in the training and validation cohorts. The time-dependent AUCs of constructed nomogram in the training cohort (A) and validation cohort (B) varying from 30 months to 60 months were plotted. [file 9219067.f1.zip › Supplementary material 2.docx]

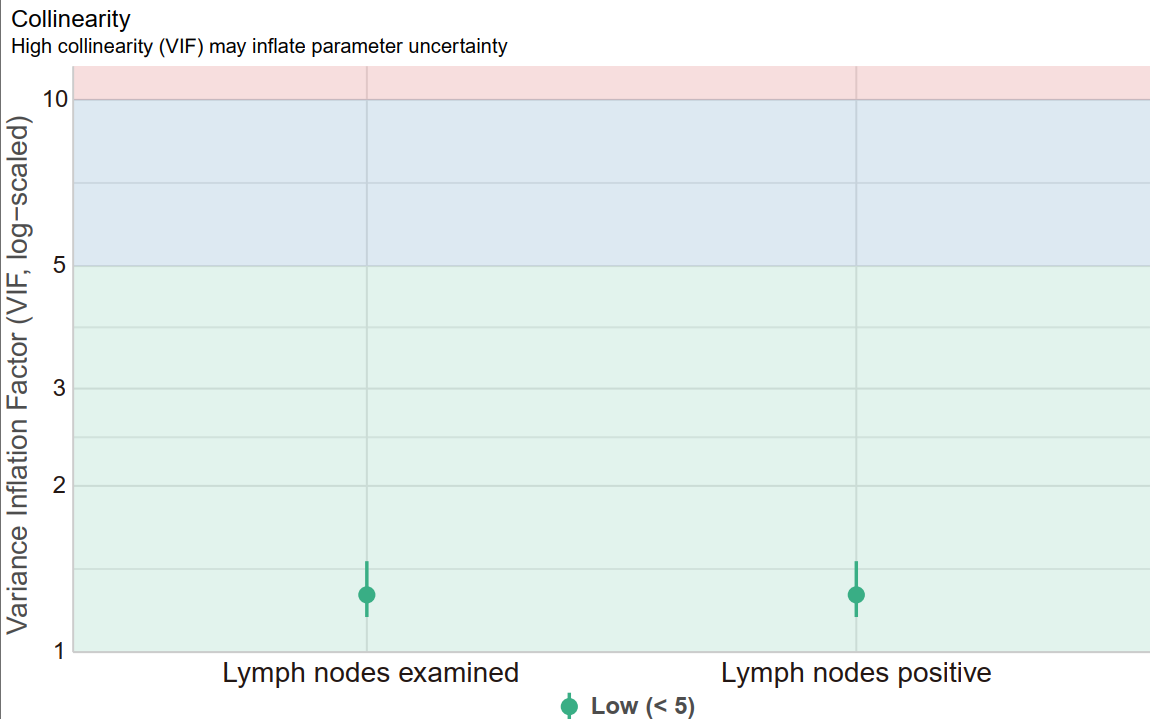


**Supplementary figure 1: VIF values for the number of lymph nodes examined and the number of lymph nodes positive.**


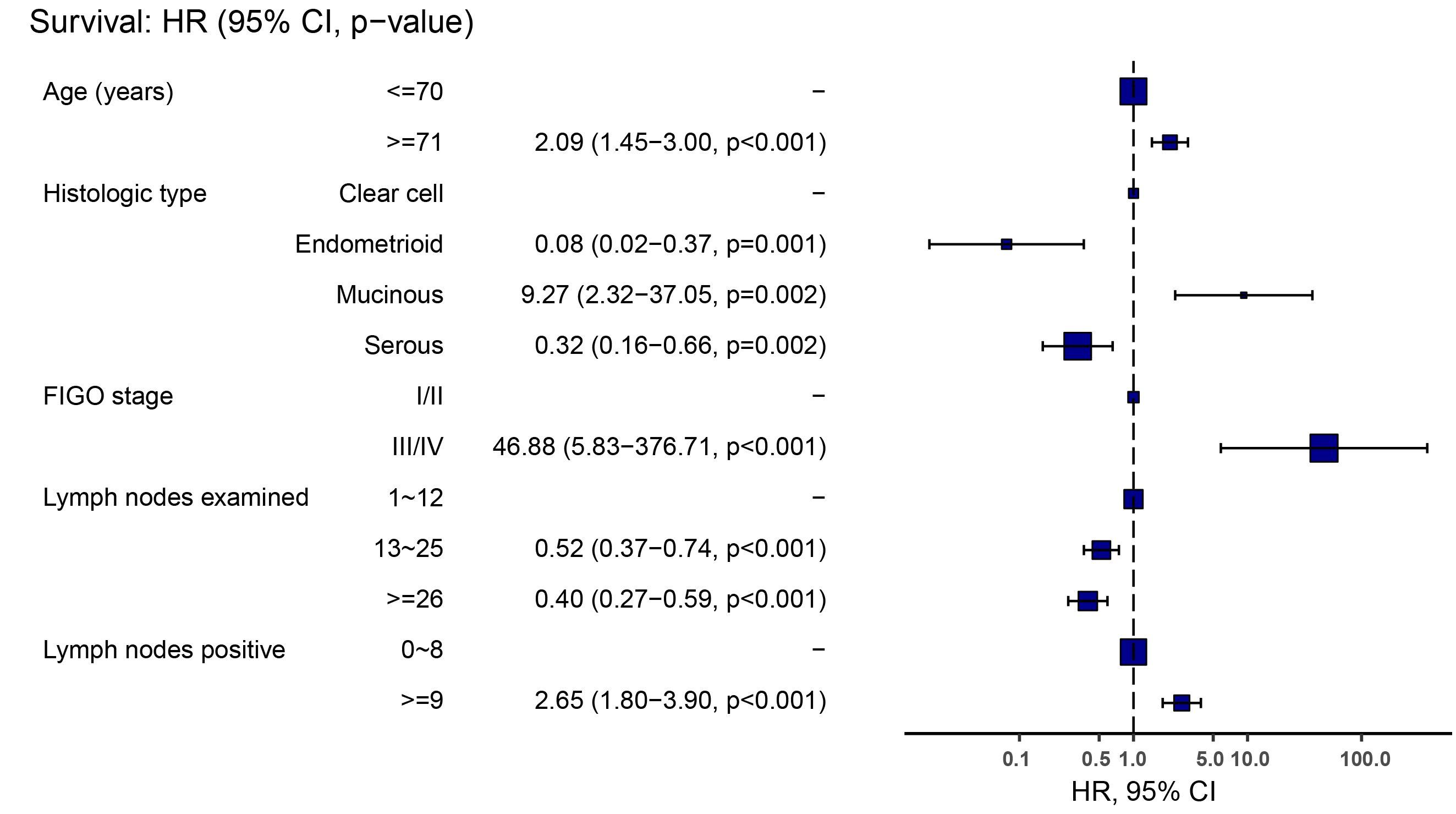


**Supplementary figure 2: Forest plot of stepwise multivariable cox regression analysis of CSS in training cohort.**


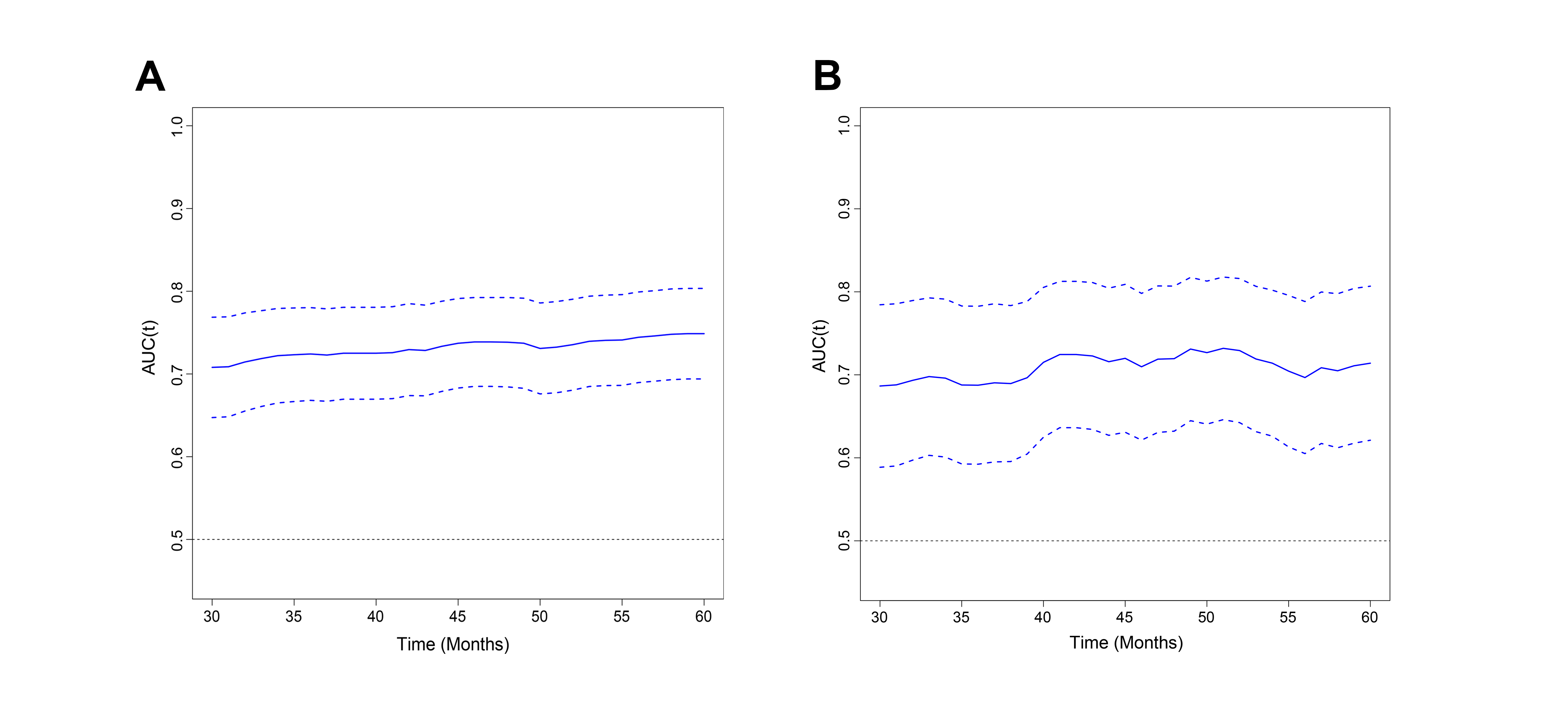


**Supplementary figure 3: AUCs varying with time of the nomogram in the training** **and validation cohort.** The time dependent AUCs of constructed nomogram in the training cohort (A) and validation cohort (B) varying from 30 months to 60 months were plotted.
